# Discontinuities in and Perceptions of Mental Health Service Path of Violent Young Offenders: A Qualitative Descriptive Study

**DOI:** 10.1007/s10597-024-01330-2

**Published:** 2024-08-01

**Authors:** Eeva Huikko, Päivi Santalahti, Terhi Aalto-Setälä, Aulikki Ahlgrén-Rimpiläinen, Riikka Lämsä

**Affiliations:** 1 Mental Health, The Equality Unit, Finnish Institute for Health and Welfare, P.O. Box 30, Helsinki, 00271 Finland; 2 Child Psychiatry, University of Turku, Turku, Finland; 3 Forensic Psychiatry, The Equality Unit, Finnish Institute for Health and Welfare, Helsinki, Finland; 4 Public Health, University of Helsinki, Helsinki, Finland

**Keywords:** Young violent offenders, Mental health services, Discontinuities, Perceptions, Qualitative

## Abstract

Studies on mental health service use among juvenile violent offenders prior to their acts of violence are sparse. Mostly, their service use seems to be short-term, although there may have been several service periods. Little is known about how they have perceived those services. Using a qualitative content analysis on data from forensic psychiatric examination statements, we studied discontinuities in the use of mental health services of 15-22-year-old violent Finnish offenders and descriptions of their perceptions of those services. There were several types of discontinuities: limited youth engagement and subsequent dropping out from services, or partial or total refusal of the proposed examinations or treatments. Most discontinuations were instigated by the youth themselves, followed by the parents and the service system. The subjects had perceived mental health services to be not beneficial for the most part, although some experienced benefits from medication. When treating children and adolescents with behavioral symptoms clinicians should identify the early signs of the process of disengagement from treatment and pay attention to the perceptions of the treatment of both the youth and their parents. Also, more research is needed on the user experience of mental health services among violent offenders, as well as factors relating to discontinuities along their mental health service path.

## Introduction

A violent offense implies serious consequences not only to the victim but also to the perpetrator, and more so in the case of juvenile offenders. If the victims survive, they may suffer from long-term psychological, physical, and social difficulties (Turanovic, 2022). In addition to the social consequences of a prison sentence, juvenile perpetrators may also suffer from perpetrator trauma due to their own violent actions (Mahlako et al., 2023; Soh et al., 2023). Psychiatric morbidity is known to be high among juvenile violent offenders, especially conduct-, personality- and substance use disorders (Elonheimo et al., 2007; Rodway et al., 2011; Hofvander et al., 2017). However, these disorders develop over a long period of time, and little is known about violent juvenile offenders’ mental health service use and treatment received during their childhood and adolescence, prior to their violent offense (Holmes et al., 2001; Sung et al., 2004). Even more sparse is knowledge about their subjective experiences of using these services.

In the Nordic countries, where healthcare systems are quite similar, previous studies have shown that during their childhood and adolescence juvenile violent offenders use mental health services, prior to their violent offense, more often than children and adolescents in general. For example, 8.5% of 7–12 -years-olds, 14.4% of 13-17-years-old and nearly one in five (18%) of 18–22-year-olds in the general population have been found to have used public outpatient or inpatient mental health services in Finland (Forsell, 2022). One third to one half of Finnish 15-18-year-old violent offenders have reportedly used mental health services of unspecified quality prior to their violent offense (Hagelstam & Häkkänen, 2006; Lindberg et al., 2016). Respectively, in a Swedish study half of 15-22-year-old violent offenders had been in psychiatric care during childhood or early adolescence (Adler et al., 1995). In a Finnish study on15-22-years-old violent offenders, of those who were reported to have had emotional/behavioral symptoms during their childhood or adolescence, three in five had used public outpatient or inpatient mental health services at the age of 7 to 15 years and three in four at the age of 16 to 22 years (Huikko et al., 2023).

As for the timing and duration of mental health service use prior to the violent offense as well as completion of treatment among young violent offenders, studies are scarce. Reportedly, the very first visit may be several years prior to a violent offense, and up to one in two have been a patient of mental health services during childhood. There have been reports of services being used for short periods but several times during childhood or adolescence. In a smaller number of cases, years of treatment contact have been reported. The use of mental health services has been rare at the time when the violent crime leading to a forensic psychiatric examination was committed. (Hagelstam & Häkkänen, 2006; Jack et al., 2015; Huikko et al., 2023.)

However, also among children and adolescents in general multiple treatment periods in mental health services and dropping out are common (de Haan et al., 2013; O’Keeffe et al., 2018; Dossett & Reid, 2020; Reid et al., 2021; Green et al., 2022). For example, when transferring from adolescent psychiatric services to adult psychiatric services, discontinuation of treatment is common, especially in the case of behavioral and substance abuse problems (Cohen et al., 2020). Dropping out from mental health services by adolescents and young adults has been found to associate with externalizing problems and disorders already from early intervention services (Farrand et al., 2009; de Haan et al., 2013; O’Keeffe et al., 2018). Co-occurrence of serious mental illness and substance use increase the likelihood of dropping out (Farrand et al., 2009; Kreyenbuhl et al., 2009; Bouchard et al., 2022). A qualitative study among adolescents with borderline personality disorders to understand the processes involved in dropping out from treatment found that the process of disengagement begins with activation of negative emotions about the treatment, e.g., the appropriateness of the treatment and therapeutic relationship. Attitudes interfering with treatment, such as splitting, hostility and experiential avoidance, arise thereafter, followed by disengagement behaviors such as irregular attendance, instrumentalized treatment, self-treatment, hiding information, and refusing the treatment. (Desrosiers et al., 2020.)

Among the general population, the perceived characteristics of the treatment and professionals seem to be more important predictors of dropping out than characteristics of the patient (de Haan et al., 2013). A mismatch with the helping professional is a common reason for the discontinuation of treatment (Green et al., 2022). For young people in general, trust and confidentiality are most important in mental health treatment (Lynch et al., 2021). The key interactional continuums concerning engagement with treatment are experiencing trust or distrust, feeling understood or misunderstood, and being treated with respect or disrespect (McCormick et al., 2023). Youth with conduct disorders as well as juvenile offenders can find it difficult to trust other people, but very little is known about how juvenile violent offenders have experienced the mental health services they received before their offence (Talia et al., 2021; Walsh et al., 2011). One qualitative British interview study on offenders in forensic mental health services aged 14 to 17 noted that services were often perceived as unhelpful (Heath & Priest, 2016). Another British interview study on 14–17 years-old offenders in general with experience of child and adolescent mental health services, found that during childhood, the future offender did not necessarily understand the reasons for using the child mental health services and felt left out of conversations between adults. They described having been afraid of mental health services before entering them and felt that medical diagnoses were stigmatizing. (Jack et al., 2015.)

In Finland, mental health services are provided for children and young people by several public bodies and are offered free of charge. The identification of mental health problems takes place in primary healthcare either at a child health clinic or in school healthcare. Both also provide early support for emerging problems. Family counselling centres provide care for mild and moderate mental health problems for people under 13 years of age, and low-threshold services provide care for young people aged 13 and over, respectively. If a young person aged 16 or over is studying at a vocational school, high school, or university, he or she will receive treatment for mild and moderate mental health problems via the educational institution’s student healthcare service. The age-limit for adult mental health services is 18–22 years, from which age, primary healthcare becomes responsible for the treatment of mild and moderate disorders. The treatment of severe mental health problems is organised in special psychiatric care services for children, adolescents, or adults. The services of private doctors and therapists supplement public services. Their users can receive part of the costs, under certain conditions, from public funds from the Social Insurance Institution of Finland (Kela).

We aimed to explore and describe the types of discontinuities in mental health service use of 33 juvenile violent offenders aged 15 to 22 years using data from their forensic psychiatric examination statements. In addition, we aimed to analyze the cited descriptions of their subjective experiences about mental health service use found in the statements.

## Sample and Methods

This study is part of the research and development of the Forensic Psychiatry 2020 program of the Finnish Institute for Health and Welfare. The data consisted of statements based on forensic psychiatric examinations. These are carried out at the request of the court if it considers a psychiatric examination of a violent offender to be necessary to weigh their criminal responsibility against not being guilty by reason of insanity. The examination is a broad-based evaluation consisting of assessments performed by a multi-professional team. In addition to the results of the examination including interviews with the subject, the statements (written by forensic psychiatrists) also contain condensed information (e.g., summaries of medical reports) obtained from various professionals (e.g., primary care nurses and doctors, psychologists, social workers, child welfare workers, teachers, nurses and doctors in child and adolescent psychiatric hospitals) working in the services which the subjects had used prior to their violent act. Therefore, it contains information on mental health symptoms, substance use, and use of public and private outpatient and public inpatient mental health services during childhood, adolescence, and early adulthood, described by the subjects, their families, and those professionals. Additionally, it includes information on family history and family relationships, peer relationships, education and support for learning, and involvement in child welfare and child welfare services during the subject’s life. The whole data of the study include the statements of the 42 youngest people (age range 15–22 years, 33 males, nine females, surveyed in forensic psychiatric examination between 2015 and 2019 (Huikko et al., 2023).

In this study we focused on analyses of the mental health service use of the 33 subjects out of 42 who reportedly had used public or private outpatient mental health services and/or public inpatient mental health services prior to a violent offense or hands on sexual offense for which a forensic psychiatric examination was requested. As part of the forensic psychiatric examination, extensive information on the offenders’ service use was gathered from professionals in various child, adolescent, and adult psychiatric units from primary to tertiary level, including health centers, student healthcare, family counselling centers, low threshold adolescent care, psychiatric emergency units, and child welfare services. The data consisted of 907 pages of text in PDF format, 18–37 (mean 27) pages/statement. The subjects’ experiences of mental health service use were cited in one third of the statements. The length of these citations was from one to a few sentences.

Since the data was textual, and the aim was to describe the phenomena related to the mental health service use without interpretations of the content, content analysis was deemed a suitable method for the analyses (Hsieh & Shannon, 2005). Basic information describing the sampling and analysis is described in an earlier study (Huikko et al., 2023). In the statements, mental health service usage was described in sentences that included various combinations of features of services illustrated in Table 1. We studied the two subthemes of mental health service usage, discontinuities of mental health service use and subjective perceptions of mental health service use. Given the data was strictly confidential and access to it was very limited, inductive coding was done by only one researcher (EH). However, the coding and analysis were guided by two senior researchers (PS and RL).

**Table 1:** Contents of mentions describing mental health service use in statements

Contents of mentions
Mental health service that provided the service
Referring mental health service if any
Who took the initiative or wrote the referral
Urgent or non-urgent need
Voluntary or involuntary examination or treatment
Symptoms or problems of the child or young person
What kind of contact (outpatient visit, inpatient care, phone contact)
What kind of examinations or treatments had been carried out (e.g., psychological examination, medication)
Duration of examinations or treatments (time, number of visits)
Discontinuities in the service use
Termination of the service usage (agreed, other)
Age of the child or adolescent when using the service
Subjective experience of the service usage

The codes and the sub-themes are presented in Table 2. For outpatient mental health services, we included visits to primary healthcare and to a private doctor’s office if the reason for the visit involved emotional or behavioral problems, and visits to a family counselling clinic and low threshold youth services and further, visits to a child, adolescent, or adult psychiatric outpatient clinic. Inpatient mental health services included periods of examinations and treatments on a child, adolescent, or adult psychiatric ward. The *descriptive information* was classified by the gender, age, psychiatric diagnoses, and educational or employment status:1. in education, training or employed 2. not in employment, education, or training.

**Table 2:** Theme, sub-themes and codes

Codes	Sub-themes	Theme
Visits to special mental health services (outpatient and/or inpatient) Visits to a family counselling clinic Visits to a low threshold youth service Visits to primary healthcare (because of emotional or behavioral problems) Visits to a private professional Treatment at a child welfare facility with psychiatric staff	Use of various mental health services	Mental health service use
A termination of an ongoing treatment or psychological examination by one’s own decision (refusal, dropout) Interruptions in or irregular use of appointments or treatments (variable treatment engagement) A planned examination or treatment which was not implemented	Type of discontinuity of mental health service use	
Discontinuity initiated by the subject Discontinuity initiated by the parent Discontinuity initiated by the helping organization Discontinuity initiated by unknown	Discontinuity initiated by whom	
Mental health use experienced as beneficial Mental health use experienced as indifferent Mental health use experienced as not beneficial Mental health use experienced as harmful	Subjective experiences of mental health service use	

In the results section we have used some translated extracts from the data. The quotations are to support the results and are mainly for illustrative purposes. They have been selected from the statements of 13 subjects. Due to the strict confidentiality of the data, we only can present quotations which secure the privacy of the subjects more than usual. We extracted any details which allow identification, and further, we do not attach any descriptions to the quotations, e.g., gender of the subject or the age that the quotation applies.

## Results

### Descriptive Information

Of the 33 subjects, 26 were males and 7 were females. The age-range was 15–22 years. None of the subjects were employed and few were engaged in education or training. In the forensic psychiatric examination, two thirds had been diagnosed with at least one personality disorder, i.e., with an F60 diagnosis (according to the International Statistical Classification of Diseases and Related Health Problems, WHO 10th edition), either as the sole or as one among several F-diagnoses. Other diagnoses included schizophrenia, schizotypal and delusional disorders (F20), mood (affective) disorders (F30), neurotic, stress-related and somatoform disorders (F40), mental retardation (F70), disorders of psychological development (F80) or behavioral and emotional disorders with onset usually occurring in childhood and adolescence (F90). Further, substance misuse was diagnosed (F10, F12, F19) as dependence or harmful in two thirds of the cases, of whom half had more than one substance misuse diagnosis. Half of the subjects had been diagnosed with both F60 and F10 -diagnoses and a third with either F 60 or F10 diagnoses.

### Discontinuities in the Mental Health Service Path

Discontinuities of service use were found among the subjects in all diagnostic categories, and most subjects (29/33, 88%) were reported to have had one or more of those. The most common case was one mention of a discontinuity in the statements based on the forensic psychiatric examinations of the subjects (12/29, 41%). However, some subjects experienced several discontinuities (up to nine) and those could be of up to six types. Most commonly, the discontinuities were instigated by the patient (Table 3).

**Table 3:** Number and percentages of subjects with discontinuities in the use of mental health services and number and percentages of mentions of discontinuities in the data classified by initiators

Initiated by	Subjects n (%) (*N* = 29)	Mentions in the data n (%) (*N* = 82)
Patient		
• Refusal of the use of mental health services	10 (30)	12 (15)
• Dropout & escape	9 (27)	19 (23)
• Refusal of using psychotropic medication	11 (33)	16 (19)
• Variable treatment engagement	9 (27)	12 (15)
Parents	8 (24)	9 (11)
Service system	4 (12)	6 (7)
Unknown	7 (21)	8 (10)
All	29 (100) a	82(100)

a Some subjects had more than one type of discontinuity in their use of mental health services

*Refusal of the use of mental health services* occurred among subjects aged 14 to 17. The most common service that was refused was the supportive contact at an adolescent psychiatric outpatient clinic, in one case in primary level services. In some cases, the subject had visited the adolescent psychiatric outpatient clinic only once and refused thereafter.

In some cases, the planned examinations were not undertaken because of the refusal. If the youth had had earlier experiences of mental health services, refusal could occur already when a service was suggested, which was the case in this example:

“The examinee was offered the opportunity to visit a doctor because of [symptoms], but the subject refused because she/he was afraid of being misdiagnosed based on her/his [symptoms].”

Another way to refuse involvement was to give false information to avoid further visits.

*Dropping out*, defined as leaving the services based on one’s own decision, was discovered both during examinations and treatment, for example during psychological examinations and support or follow-up visits to a psychiatric nurse or doctor. Dropping out occurred most often from adolescent psychiatric services but also from adult psychiatric and primary level services, the last being illustrated in the following:

“The examinee visited a psychiatric nurse twice before dropping out of treatment contact.”

*Escaping* happened in the case of both voluntary and involuntary adolescent psychiatric wards, once or several times.

Subjects escaping from inpatient services were aged 13 to 17. The descriptions often included the manner of escape and the time which had elapsed from the access to the ward, which was sometimes only hours.

Eventually, escaping occasionally ended the involvement with mental health services.

*Refusal to use psychotropic medication* was found in cases from 12 years of age. In such cases the youth did not accept the suggested or prescribed psychotropic medication. Refusal of the use of a medicine could appear later, when the youth stopped it on their own volition, which was the case in this example:

“With increasing [symptoms] [a medicine] had been prescribed to the examinee in [season, year]. On her/his own initiative, the examinee decided to terminate the medication [month, same year].”

*Variable treatment engagement* took the form of irregular use of medication and poor attendance to appointments in adolescent psychiatry or low threshold services, and was found during both shorter and longer treatments as illustrated in the following two examples:

“[Month-month/year] the examinee had attended three out of nine appointments in the adolescent psychiatric outpatient clinic.”

“In [year - year], appointments with a psychologist were arranged for the examinee, who occasionally attended irregularly.”

In addition, a non-used scheduled acute appointment was sometimes soon followed by the use of emergency services and some subjects who had escaped from inpatient treatment later returned to the ward voluntarily.

In some cases, the discontinuation of the mental health service use was instigated by the parents (Table 3). Among these, parental refusal for their child’s examinations was the most common. Some parents had refused examinations in primary or special care proposed by the primary care workers for their child. Some parents had cancelled the first appointment at adolescent psychiatric outpatient clinics arranged based on primary care referral, as described according to one statement:

“The examinee was referred to the adolescent psychiatric outpatient clinic, but the examinee’s [parent] later cancelled the appointment.”

During psychiatric treatment, irregular implementation of the child’s medication and unilateral parental decisions to terminate the treatment were mentioned, as described in an example of the latter from the data:

“The treatment plan was for parental support visits, of which only [number of visits] was realized as the parents found them unnecessary.”

During childhood, treatment discontinuities initiated by the parents were more common than those instigated by the child.

In a few cases, discontinuities in treatment resulting from the functioning of the mental health service system were identified, such as a delay of over one year before access to the planned next service or a lack of funding for planned psychotherapy (Table 3). In addition, there were some mentions of recommended examinations or referrals to examinations or treatment but no mentions of implementing them or the reasons for them not being implemented. In one statement the following conclusion was cited:

“Based on the inpatient examination, it had been found that the examinee is still in need of long-term child psychiatric help.”

However, we did not find any mentions of treatment of the subject until some years later.

Sometimes the interruption in treatment was a consequence of a decision made in child welfare services, such as a change in the placement of a youth in custody. In addition, child welfare services had discontinued the treatment of a youth by shifting the whole responsibility for their psychiatric treatment from a hospital outpatient clinic to a private psychiatrist, which meant that the youth had to start from the beginning and build a new relationship and trust with a new psychiatrist.

### Subjective Experience of Mental Health Services and Substance use

11 subjects (38%) had reportedly described their subjective experiences of mental health service use. Beneficial experiences had mostly been related to medication. Some subjects with asocial or emotionally unstable personality disorder (F60) or early onset behavioral and emotional disorder (F90) had found that the drugs prescribed for concentration difficulties or impulsivity were useful. For example, a description in one statement explained:

“In primary healthcare, the examinee had received an ADHD diagnosis and the medication was methylphenidate, which she/he felt had benefited her/him.”

There was also a mention of a beneficial supportive outpatient intervention. In addition, the use of mental health services was mentioned as beneficial in terms of avoiding difficulties with other authorities.

However, mental health services in whole or in part had been experienced also as not beneficial, and were described as useless, or the patient not benefitting from the treatment offered, not getting the help needed, or that the medication prescribed by a doctor had minimal benefit. The following example illustrates how the subject did not find the treatment helpful as a whole, nor the medication as part of it:

“The examinee felt that the treatment periods had been in vain. […] The examinee had not experienced any help from [the medicine].”

Treatments received during childhood had been reported as confusing and incomprehensive, in cases when the subject had not understood the discussions during the treatment sessions, for example. Additionally, bad experiences with doctors and nurses had been mentioned, which resulted in the reluctance to use any services. A few had experienced their treatment to be harmful, for example some patients with schizophrenia, or schizotypal or delusional disorder (F20), had felt that the medication was harmful, for example:

“The examinee felt that the medicine may have made her/him violent.”

The experience of a few was indifferent: mental health services were considered to be unnecessary, or other social contacts more important, as mentioned in one statement as the reason for the termination of treatment:

“[…] treatment contacts were interrupted because the examinee did not arrive at the scheduled times. The subject said that it was more important for them to spend time with friends.”

## Discussion

To the best of our knowledge, our study is the first to describe discontinuities in the use of mental health services among juvenile violent offenders during childhood and adolescence prior to a violent act that led to a trial. We also describe their subjective experiences of mental health services as cited in the forensic psychiatric examination statements. Our main finding was that discontinuities had occurred in the lifelong mental health service path of most subjects and there were several types of discontinuities: varying degrees of collaboration, dropping out from services, or partial or total refusal of the proposed examinations or treatments. Most discontinuities in the service or treatment were initiated by an adolescent. However, parents and the service system also instigated some discontinuities. In addition, examinations and treatments in some cases were not implemented for unknown reasons. The second main finding was that the subjects had perceived the mental health services received to be not beneficial for the most part, but also some benefits had been mentioned.

Although we found discontinuities among the subjects in all diagnostic categories, the majority of the subjects had been diagnosed with either a personality disorder or a substance abuse disorder diagnosis, or both. We found many of the disengagement behaviors described earlier among adolescents with borderline personality disorder (Desrosiers et al., 2020). We also found active refusal of treatment, which is common among youth with asocial attitudes (Kapoor et al., 2018). However, some descriptions of not using services in our study did not differ from youth in general, such as prioritizing peer relationships at the expense of treatment (e.g., Green et al., 2022). Irregular participation in treatment has also been considered characteristic of young people in general (Gilbert et al., 2012; Dewhurst et al., 2017). Disengagement from the treatment most commonly occurs early, but professionals do not necessarily notice negative emotions at the outset of this process (Farrand et al., 2009; Kreyenbuhl et al., 2009; Desrosiers et al., 2020). In general, negative experiences with mental health services reduce adolescents’ willingness to continue using them (Platell et al., 2020). Many of our subjects had reportedly experienced the use of mental health services not to be beneficial, which has also been described for youth with conduct disorders and concentration difficulties (Platell et al., 2021). Our findings suggest that when treating children and adolescents with behavioral symptoms, it is important to identify the early signs of the process of disengagement from treatment, such as lack of trust, and to promote an open discussion with the patient and the family about the topic and their expectations for treatment.

We found that parents instigated discontinuities in treatment for some subjects, especially when further examinations had been recommended for their child. Since treatment of the mental health problems of minors in public services is free of charge for families in Finland, the costs were unlikely to affect the parents’ decision. At this stage of the mental health treatment process, a fear of social stigma and labeling of the child can be among parental barriers to accessing mental health treatment of a child (Reardon et al., 2017). The experienced benefit of the treatment is related to the continuation of the treatment, and dropping out during the treatment can reflect a perception of few benefits (Horwitz et al., 2012). When treating children and adolescents with behavioral symptoms or disorders, building open and respectful care cooperation with the parents seems to be central to the continuity of the care.

Some discontinuities were initiated by the service system. The use of mental health services by some subjects ended due to child welfare actions. Discontinuities in the treatment instigated by the mental health service system included a delay or lack of planned services. In earlier studies, treatment interruptions by professionals involved, against the will of the youth have been reported (Green et al., 2022). Mental health service processes as such have also been mentioned among the barriers to service use (Yonek et al., 2019; Roberts et al., 2022). In addition, there are structural discontinuities in the mental health service system, such as the age-based transition to adult services, and the transition from assessment to treatment, which often implies a change of the responsible professionals (Luk et al., 2001; Green et al., 2022; de Soet et al., 2023). Moreover, differences in patients’ dropping out depend on the organization delivering the service on the one hand, and the practitioner on the other (Edbrooke-Childs et al., 2020). Open-minded evaluation and research of the service system would be needed to better ascertain how and when these service system discontinuities occur.

### Strengths and Limitations

The main strength of our study is the life-long data on the use of mental health services of young violent offenders. The data coverage was good, since it included all 33 written statements in the national archive of forensic psychiatric examinations during the years 2015–2019 that concerned the youngest violent offenders who had received mental health services. Therefore, the results represent young violent offenders fairly well, but do not represent the juvenile offender population in general. The data is based on original case documents from all levels of the mental health service system. A further strength lies in our qualitative approach, which was able to examine all of the qualities of the phenomenon. There are also many limitations. The major limitations are the small sample, the secondary and retrospective nature of the data, and the fact that the data was coded by only one author. A further limitation is that the original clinical data submitted for forensic psychiatric examination were processed by a number of different professionals. Therefore, the reliability of the retrospective information in the statements may vary. The subjects were not interviewed by the authors, and further, the analysis of the subjective experiences of mental health service use was based on secondary sources and was not cited in all statements. The primary task of the forensic psychiatric examination and the clinical situation of the subjects may have influenced the content of the information provided by the subjects. However, this study makes a noteworthy contribution to the scarce knowledge of mental health service use history of violent offenders prior to committing a violent act.

## Conclusions

In conclusion, the mental health treatment path of juvenile violent offenders is prone to several types of discontinuities. To increase the commitment to treatment of children and adolescents with behavioral and emotional symptoms, the service system should pay attention to both their perception of the treatment and their parents’ perceptions of it. Building open and respectful care cooperation with parents seems to be central to the continuity of the care of children and adolescents with behavioral symptoms. It is crucial to identify the early signs of the process of disengagement from treatment and to promote open discussion with the patient and the family about the topic and their expectations for treatment. More longitudinal research on mental health service paths is needed, including studies on how services are provided. These analyses would benefit from combining them with qualitative interviews with the users themselves. Discontinuities in treatment caused by child welfare actions should be noticed and analyzed further.

## Data Availability

All data relevant to the study are included in the article. Due to the strict confidentiality orders concerning the data, presenting data samples is limited. Additional information about the availability of the data can be obtained from Findata.
